# 4-(3-Fluoro­phen­yl)-1-(2-oxoindolin-3-yl­idene)thio­semicarbazide

**DOI:** 10.1107/S1600536810034951

**Published:** 2010-09-04

**Authors:** Muhammad Ramzan, Humayun Pervez, M. Nawaz Tahir, Muhammad Yaqub

**Affiliations:** a Department of Chemistry, Bahauddin Zakariya University, Multan 60800, Pakistan; b Department of Physics, University of Sargodha, Sargodha, Pakistan

## Abstract

In the title compound, C_15_H_11_FN_4_OS, there are three independent mol­ecules, each with a disordered 3-fluoro­phenyl group [occupancy ratios = 0.547 (17):0.453 (17), 0.645 (5):0.355 (5) and 0.626 (15):0.374 (15)] and displaying dihedral angles of 4.2 (3), 25.2 (6) and 32.4 (5)° between the 2-oxoindoline and fluoro-substituted phenyl rings. Strong intra­molecular N—H⋯N and N—H⋯O and weak intra­molecular C—H⋯S hydrogen bonds complete *S*(5) and *S*(6) ring motifs, while strong inter­molecular N—H⋯O hydrogen bonds inter­connect the three independent mol­ecules through *R*_3_^3^(12) ring motifs. The three-mol­ecule units are in turn linked into polymeric sheets *via* C—H⋯F and C—H⋯S hydrogen bonds and π–π inter­actions [centroid–centroid distances in the range 3.520 (2)–3.820 (9) Å].

## Related literature

For our work on the synthesis of biologically important isatin (systematic name 1*H*-indole-2,3-dione) derivatives, see: Pervez *et al.* (2007 ▶, 2008 ▶, 2009 ▶). For related structures, see: Pervez *et al.* (2010 ▶). For graph-set notation, see: Bernstein *et al.* (1995 ▶).
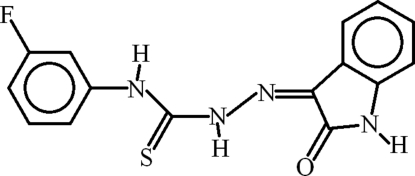


## Experimental

### 

#### Crystal data

C_15_H_11_FN_4_OS
*M* *_r_* = 314.34
Triclinic, 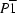

*a* = 8.0582 (3) Å
*b* = 14.3640 (5) Å
*c* = 19.4456 (8) Å
α = 86.338 (2)°
β = 89.299 (1)°
γ = 75.128 (2)°
*V* = 2170.93 (14) Å^3^
*Z* = 6
Mo *K*α radiation
μ = 0.24 mm^−1^
*T* = 296 K
0.28 × 0.22 × 0.20 mm

#### Data collection

Bruker Kappa APEXII CCD diffractometer
Absorption correction: multi-scan (*SADABS*; Bruker, 2005 ▶) *T*_min_ = 0.942, *T*_max_ = 0.950
31845 measured reflections
7851 independent reflections
4225 reflections with *I* > 2σ(*I*)
*R*_int_ = 0.059

#### Refinement

*R*[*F* ^2^ > 2σ(*F* ^2^)] = 0.063
*wR*(*F* ^2^) = 0.178
*S* = 1.02
7851 reflections
573 parameters
3 restraints
H-atom parameters constrained
Δρ_max_ = 0.31 e Å^−3^
Δρ_min_ = −0.30 e Å^−3^

### 

Data collection: *APEX2* (Bruker, 2009 ▶); cell refinement: *SAINT* (Bruker, 2009 ▶); data reduction: *SAINT*; program(s) used to solve structure: *SHELXS97* (Sheldrick, 2008 ▶); program(s) used to refine structure: *SHELXL97* (Sheldrick, 2008 ▶); molecular graphics: *ORTEP-3 for Windows* (Farrugia, 1997 ▶) and *PLATON* (Spek, 2009 ▶); software used to prepare material for publication: *WinGX* (Farrugia, 1999 ▶) and *PLATON*.

## Supplementary Material

Crystal structure: contains datablocks global, I. DOI: 10.1107/S1600536810034951/bg2363sup1.cif

Structure factors: contains datablocks I. DOI: 10.1107/S1600536810034951/bg2363Isup2.hkl

Additional supplementary materials:  crystallographic information; 3D view; checkCIF report

## Figures and Tables

**Table 1:** Hydrogen-bond geometry (Å, °)

*D*—H⋯*A*	*D*—H	H⋯*A*	*D*⋯*A*	*D*—H⋯*A*
N1—H1⋯O3^i^	0.86	2.03	2.837 (3)	155
N3—H3*A*⋯O1	0.86	2.11	2.778 (4)	134
N4—H4*A*⋯N2	0.86	2.13	2.596 (4)	114
N5—H5*A*⋯O1^ii^	0.86	2.05	2.881 (4)	164
N7—H7⋯O2	0.86	2.11	2.785 (4)	135
N8—H8⋯N6	0.86	2.14	2.599 (4)	113
N9—H9⋯O2^iii^	0.86	2.06	2.871 (3)	157
N11—H11*C*⋯O3	0.86	2.09	2.767 (3)	135
N12—H12*C*⋯N10	0.86	2.13	2.590 (4)	113
C4—H4⋯F2*A*^iv^	0.93	2.48	3.215 (7)	136
C15*A*—H15*A*⋯S1	0.93	2.57	3.223 (18)	127
C30*A*—H30*A*⋯S2	0.93	2.54	3.208 (6)	129
C42*A*—H42*A*⋯S2^v^	0.93	2.87	3.763 (12)	162
C45*A*—H45*A*⋯S3	0.93	2.72	3.260 (13)	118

Symmetry codes: (i) 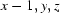; (ii) 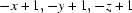; (iii) 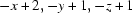; (iv) 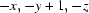; (v) 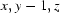.

**Fig. 1.**
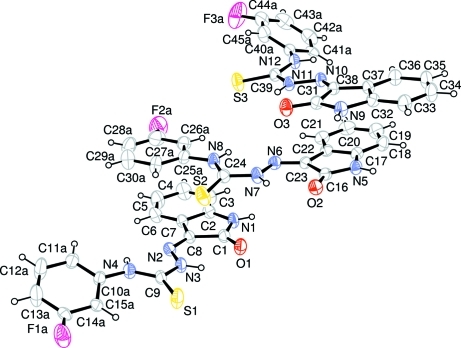
View of the title compound with the atom numbering scheme. The thermal ellipsoids are drawn at the 30% probability level. H-atoms are shown by small circles of arbitrary radii.

**Fig. 2.**
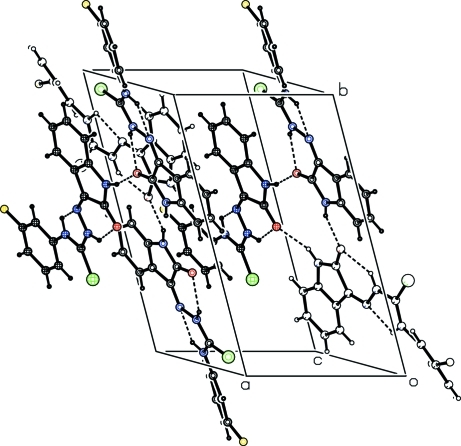
The partial packing (PLATON; Spek, 2009) which shows that molecules form polymeric sheets with central R33(12) ring motifs. The H-atoms not involved in H-bondings have been omitted for clarity.

## supplementary crystallographic information

### Comment

In continuation of our work on the synthesis of biologically important isatin derivatives (Pervez *et al.*, 2007, 2008, 2009), we report herein the structure and preparation of the title compound (I, Fig. 1). When compared with the crystal structure of *i.e.* 4-(2-fluorophenyl)-1-(2-oxoindolin-3-ylidene) thiosemicarbazide (II) (Pervez *et al.*, 2010), it can be noticed they differ in the position of the fluoro function at the phenyl ring bound to N^4^ in the thiosemicarbazone moiety.

There are three independent molecules in (I). The 3-fluorophenyl moiety is disordered in each one with different site occupancy ratios (0.547 (17):0.453 (17), 0.645 (5):0.355 (5) and 0.626 (15):0.374 (15), respectively. In all three molecules, the 2-oxoindolin A (C1–C8/N1/O1), B (C16–C23/N5/O2) and C (C31–C38/N9/O3) groups are planar with r. m. s. deviations of 0.0054, 0.0100 and 0.0036 Å, respectively. The thiosemicarbazide D (N2/N3/C9/S1/N4), E (N6/N7/C24/S2/N8) and F (N10/N11/C39/S3/N12) groups, in turn, are also planar with r. m. s. deviations of 0.0060, 0.0336 and 0.0192 Å, respectively. The dihedral angles between A/D, B/E, C/F are 9.24 (15), 7.67 (14), 6.13 (17).

Due to strong intramolecular H-bondings of N—H···N and N—H···O type, there exist one S(5) and one S(6) (Bernstein *et al.*, 1995) ring motif, respectively (Table 1, Fig. 2). A weak intramolecular C—H···S H-bond is also present in each molecule (Table 1). The three molecules are interconnected through strong intermolecular H-bonds of N—H···O type (Table 1, Fig. 2) and complete *R*_3_^3^(12) ring motifs. The molecules are stabilized in the form of polymeric sheets which are finally formed due to C—H···F and C—H···S interactions (Table 1, Fig. 2). In the stabilization of molecules, π–π interactions present play a relevant role.

### Experimental

To a hot solution of isatin (0.74 g, 5.0 mmol) in ethanol (10 ml) containing a few drops of glacial acetic acid was added 4-(3-fluorophenyl)thiosemicarbazide (0.93 g, 5.0 mmol) dissolved in ethanol (10 ml) under stirring. The reaction mixture was then heated under reflux for 2 h. The yellow crystalline solid formed during heating was collected by suction filtration. Thorough washing with hot ethanol followed by ether afforded the target compound (I) in pure form (1.12 g, 71%), m. p. 513 K (*d*). The single crystals of (I) were grown in acetone-ethanol (1:4) by diffusion method at room temperature.

### Refinement

The 3-fluorophenyl moiety is disordered in all three independent structures and their phenyl rings have been fitted into regular hexagons (C···C = 1.39Å.). The occupancy ratio of disordered groups is 0.547 (17):0.453 (17), 0.645 (5):0.355 (5) and 0.626 (15):0.374 (15), respectively.

The H-atoms were positioned geometrically (N—H = 0.86, C–H = 0.93 Å) and refined as riding with *U*_iso_(H) = *xU*_eq_(C, N), where *x* = 1.2 for all H-atoms.

### Crystal data

**Table:**

C_15_H_11_FN_4_OS	*Z* = 6
*M**_r_* = 314.34	*F*(000) = 972
Triclinic, *P*1	*D*_x_ = 1.443 Mg m^−^^3^
Hall symbol: -P 1	Mo *K*α radiation, λ = 0.71073 Å
*a* = 8.0582 (3) Å	Cell parameters from 4225 reflections
*b* = 14.3640 (5) Å	θ = 2.1–25.3°
*c* = 19.4456 (8) Å	µ = 0.24 mm^−^^1^
α = 86.338 (2)°	*T* = 296 K
β = 89.299 (1)°	Prisms, yellow
γ = 75.128 (2)°	0.28 × 0.22 × 0.20 mm
*V* = 2170.93 (14) Å^3^	

### Data collection

**Table:**

Bruker Kappa APEXII CCD diffractometer	7851 independent reflections
Radiation source: fine-focus sealed tube	4225 reflections with *I* > 2σ(*I*)
graphite	*R*_int_ = 0.059
Detector resolution: 8.10 pixels mm^-1^	θ_max_ = 25.3°, θ_min_ = 2.1°
ω scans	*h* = −9→9
Absorption correction: multi-scan (*SADABS*; Bruker, 2005)	*k* = −16→17
*T*_min_ = 0.942, *T*_max_ = 0.950	*l* = −23→23
31845 measured reflections	

### Refinement

**Table:**

Refinement on *F*^2^	Secondary atom site location: difference Fourier map
Least-squares matrix: full	Hydrogen site location: inferred from neighbouring sites
*R*[*F*^2^ > 2σ(*F*^2^)] = 0.063	H-atom parameters constrained
*wR*(*F*^2^) = 0.178	*w* = 1/[σ^2^(*F*_o_^2^) + (0.0739*P*)^2^ + 0.9447*P*] where *P* = (*F*_o_^2^ + 2*F*_c_^2^)/3
*S* = 1.02	(Δ/σ)_max_ = 0.001
7851 reflections	Δρ_max_ = 0.31 e Å^−^^3^
573 parameters	Δρ_min_ = −0.30 e Å^−^^3^
3 restraints	Extinction correction: *SHELXL97* (Sheldrick, 2008), Fc^*^=kFc[1+0.001xFc^2^λ^3^/sin(2θ)]^-1/4^
Primary atom site location: structure-invariant direct methods	Extinction coefficient: 0.0055 (13)

### Special details

**Table:**

Geometry. Bond distances, angles *etc*. have been calculated using the rounded fractional coordinates. All su's are estimated from the variances of the (full) variance-covariance matrix. The cell e.s.d.'s are taken into account in the estimation of distances, angles and torsion angles
Refinement. Refinement of *F*^2^ against ALL reflections. The weighted *R*-factor *wR* and goodness of fit *S* are based on *F*^2^, conventional *R*-factors *R* are based on *F*, with *F* set to zero for negative *F*^2^. The threshold expression of *F*^2^ > σ(*F*^2^) is used only for calculating *R*-factors(gt) *etc*. and is not relevant to the choice of reflections for refinement. *R*-factors based on *F*^2^ are statistically about twice as large as those based on *F*, and *R*- factors based on ALL data will be even larger.

### Fractional atomic coordinates and isotropic or equivalent isotropic displacement parameters (Å^2^)

**Table:**

	*x*	*y*	*z*	*U*_iso_*/*U*_eq_	Occ. (<1)
S1	0.26523 (19)	0.98975 (8)	0.34158 (6)	0.0868 (5)	
F1A	0.286 (9)	1.302 (7)	0.217 (8)	0.109 (6)	0.547 (17)
O1	0.1679 (4)	0.68968 (18)	0.35500 (15)	0.0677 (11)	
N1	0.0699 (4)	0.5919 (2)	0.28535 (17)	0.0604 (11)	
N2	0.1031 (4)	0.8231 (2)	0.22575 (16)	0.0538 (11)	
N3	0.1585 (4)	0.8620 (2)	0.27979 (16)	0.0576 (11)	
N4	0.1882 (4)	0.9854 (2)	0.20740 (16)	0.0596 (11)	
C1	0.1113 (5)	0.6730 (3)	0.3007 (2)	0.0543 (16)	
C2	0.0125 (5)	0.5970 (3)	0.2173 (2)	0.0567 (16)	
C3	−0.0410 (5)	0.5294 (3)	0.1832 (3)	0.0733 (19)	
C4	−0.0925 (6)	0.5520 (3)	0.1160 (3)	0.086 (2)	
C5	−0.0896 (6)	0.6393 (3)	0.0833 (3)	0.0860 (19)	
C6	−0.0350 (5)	0.7081 (3)	0.1178 (2)	0.0703 (16)	
C7	0.0155 (5)	0.6861 (3)	0.1854 (2)	0.0524 (14)	
C8	0.0788 (4)	0.7381 (2)	0.23583 (19)	0.0501 (12)	
C9	0.2032 (5)	0.9476 (2)	0.27259 (19)	0.0532 (16)	
C10A	0.2262 (19)	1.0704 (11)	0.1760 (10)	0.0534 (18)	0.547 (17)
C11A	0.246 (2)	1.0776 (12)	0.1049 (10)	0.061 (4)	0.547 (17)
C12A	0.263 (2)	1.1637 (14)	0.0722 (9)	0.103 (4)	0.547 (17)
C13A	0.2589 (19)	1.2426 (11)	0.1105 (10)	0.095 (3)	0.547 (17)
C14A	0.2389 (18)	1.2354 (11)	0.1816 (10)	0.047 (4)	0.547 (17)
C15A	0.2225 (18)	1.1493 (13)	0.2143 (9)	0.047 (3)	0.547 (17)
C13B	0.272 (2)	1.2310 (14)	0.1052 (13)	0.095 (3)	0.453 (17)
C14B	0.3003 (18)	1.2194 (13)	0.1760 (13)	0.047 (4)	0.453 (17)
C15B	0.267 (2)	1.1406 (16)	0.2134 (11)	0.047 (3)	0.453 (17)
C11B	0.178 (2)	1.0849 (16)	0.1092 (13)	0.061 (4)	0.453 (17)
F1B	0.285 (11)	1.299 (9)	0.212 (10)	0.109 (6)	0.453 (17)
C10B	0.206 (2)	1.0733 (14)	0.1800 (13)	0.0534 (18)	0.453 (17)
C12B	0.211 (3)	1.1638 (19)	0.0718 (11)	0.103 (4)	0.453 (17)
S2	0.57526 (16)	0.71997 (8)	0.27758 (6)	0.0787 (5)	
F2A	0.2702 (6)	0.4925 (3)	0.0210 (2)	0.141 (2)	0.645 (5)
O2	0.7099 (3)	0.51552 (17)	0.47192 (12)	0.0573 (10)	
N5	0.6913 (4)	0.3622 (2)	0.50827 (15)	0.0536 (11)	
N6	0.5561 (4)	0.4643 (2)	0.34248 (16)	0.0518 (11)	
N7	0.5872 (4)	0.5516 (2)	0.33703 (16)	0.0582 (11)	
N8	0.5036 (4)	0.5648 (2)	0.22516 (16)	0.0603 (11)	
C16	0.6704 (4)	0.4392 (3)	0.46365 (19)	0.0476 (12)	
C17	0.6293 (4)	0.2887 (3)	0.48129 (19)	0.0496 (14)	
C18	0.6306 (5)	0.1991 (3)	0.5113 (2)	0.0643 (17)	
C19	0.5656 (6)	0.1399 (3)	0.4723 (3)	0.0753 (19)	
C20	0.5044 (5)	0.1674 (3)	0.4063 (3)	0.0730 (19)	
C21	0.5038 (5)	0.2575 (3)	0.3766 (2)	0.0590 (16)	
C22	0.5672 (4)	0.3181 (3)	0.41468 (19)	0.0480 (12)	
C23	0.5922 (4)	0.4138 (2)	0.40045 (18)	0.0448 (12)	
C24	0.5525 (5)	0.6092 (3)	0.2761 (2)	0.0546 (14)	
C25A	0.4523 (6)	0.5959 (3)	0.15770 (19)	0.0605 (17)	0.645 (5)
C26A	0.3903 (5)	0.5317 (3)	0.12122 (16)	0.0715 (17)	0.645 (5)
C27A	0.3384 (4)	0.5553 (3)	0.05302 (16)	0.093 (2)	0.645 (5)
C28A	0.3485 (7)	0.6432 (3)	0.0213 (2)	0.113 (3)	0.645 (5)
C29A	0.4105 (9)	0.7074 (3)	0.0578 (3)	0.112 (3)	0.645 (5)
C30A	0.4624 (7)	0.6837 (3)	0.1260 (3)	0.087 (2)	0.645 (5)
C28B	0.3502 (8)	0.64587 (14)	0.0212 (2)	0.113 (3)	0.355 (5)
C29B	0.4134 (9)	0.70940 (11)	0.0579 (2)	0.112 (3)	0.355 (5)
C30B	0.4662 (8)	0.68490 (10)	0.1259 (2)	0.087 (2)	0.355 (5)
C26B	0.3926 (4)	0.53333 (17)	0.12057 (16)	0.0715 (17)	0.355 (5)
F2B	0.4266 (11)	0.79699 (12)	0.0362 (3)	0.141 (2)	0.355 (5)
C25B	0.4558 (5)	0.59686 (14)	0.15726 (19)	0.0605 (17)	0.355 (5)
C27B	0.3398 (5)	0.55784 (16)	0.05254 (18)	0.093 (2)	0.355 (5)
S3	0.73951 (16)	0.32405 (7)	0.17558 (5)	0.0755 (5)	
F3A	0.683 (2)	0.0464 (13)	0.0360 (7)	0.164 (6)	0.626 (15)
O3	1.0173 (2)	0.42029 (11)	0.35189 (7)	0.0627 (10)	
N9	1.1234 (3)	0.36096 (15)	0.46050 (8)	0.0523 (11)	
N10	0.9445 (4)	0.2242 (2)	0.35231 (15)	0.0479 (11)	
N11	0.8890 (4)	0.2813 (2)	0.29489 (15)	0.0504 (11)	
N12	0.8135 (4)	0.1556 (2)	0.25218 (15)	0.0553 (11)	
C31	1.0500 (4)	0.3568 (3)	0.3997 (2)	0.0491 (12)	
C32	1.1409 (4)	0.2743 (3)	0.50170 (19)	0.0474 (12)	
C33	1.2072 (5)	0.2516 (3)	0.5665 (2)	0.0618 (17)	
C34	1.2097 (6)	0.1617 (3)	0.5956 (2)	0.0728 (17)	
C35	1.1458 (6)	0.0964 (3)	0.5617 (2)	0.0702 (16)	
C36	1.0786 (5)	0.1204 (3)	0.4960 (2)	0.0585 (16)	
C37	1.0759 (4)	0.2105 (2)	0.46541 (18)	0.0451 (12)	
C38	1.0161 (4)	0.2592 (2)	0.39974 (18)	0.0443 (12)	
C39	0.8147 (4)	0.2479 (3)	0.24190 (18)	0.0495 (12)	
C40A	0.7514 (15)	0.0966 (8)	0.2117 (7)	0.0510 (19)	0.626 (15)
C41A	0.6868 (14)	0.0235 (9)	0.2428 (6)	0.051 (3)	0.626 (15)
C42A	0.6494 (15)	−0.0458 (8)	0.2032 (8)	0.087 (3)	0.626 (15)
C43A	0.6766 (16)	−0.0420 (10)	0.1324 (8)	0.096 (3)	0.626 (15)
C44A	0.7412 (16)	0.0311 (12)	0.1013 (6)	0.080 (4)	0.626 (15)
C45A	0.7786 (16)	0.1004 (9)	0.1409 (7)	0.079 (4)	0.626 (15)
C43B	0.641 (3)	−0.0376 (18)	0.1348 (14)	0.096 (3)	0.374 (15)
C44B	0.664 (3)	0.047 (2)	0.1009 (11)	0.080 (4)	0.374 (15)
C45B	0.729 (3)	0.1105 (16)	0.1364 (13)	0.079 (4)	0.374 (15)
C41B	0.749 (2)	0.0064 (15)	0.2397 (10)	0.051 (3)	0.374 (15)
F3B	0.782 (4)	0.046 (2)	0.0301 (12)	0.164 (6)	0.374 (15)
C40B	0.771 (3)	0.0904 (15)	0.2058 (13)	0.0510 (19)	0.374 (15)
C42B	0.684 (3)	−0.0576 (14)	0.2042 (14)	0.087 (3)	0.374 (15)
H4	−0.13029	0.50738	0.09189	0.1033*	
H12A	0.27593	1.16848	0.02460	0.1229*	0.547 (17)
H13A	0.26982	1.30018	0.08863	0.1141*	0.547 (17)
H15A	0.20914	1.14453	0.26189	0.0564*	0.547 (17)
H4A	0.14832	0.95257	0.17929	0.0714*	
H5	−0.12456	0.65252	0.03751	0.1029*	
H6	−0.03279	0.76712	0.09572	0.0840*	
H11A	0.24864	1.02481	0.07922	0.0737*	0.547 (17)
H1	0.07771	0.54271	0.31394	0.0724*	
H3	−0.04221	0.46996	0.20489	0.0877*	
H3A	0.16572	0.83200	0.31979	0.0690*	
H11B	0.13650	1.03989	0.08691	0.0737*	0.453 (17)
H12B	0.19239	1.17151	0.02446	0.1229*	0.453 (17)
H13B	0.29483	1.28376	0.08015	0.1141*	0.453 (17)
H15B	0.28549	1.13279	0.26075	0.0564*	0.453 (17)
H18	0.67319	0.17959	0.55553	0.0771*	
H28A	0.31381	0.65903	−0.02433	0.1356*	0.645 (5)
H29A	0.41731	0.76618	0.03655	0.1343*	0.645 (5)
H30A	0.50390	0.72670	0.15038	0.1040*	0.645 (5)
H19	0.56280	0.07915	0.49123	0.0901*	
H20	0.46299	0.12487	0.38146	0.0875*	
H21	0.46179	0.27647	0.33220	0.0708*	
H26A	0.38350	0.47289	0.14244	0.0855*	0.645 (5)
H5A	0.73701	0.35805	0.54846	0.0642*	
H7	0.62914	0.57234	0.37167	0.0700*	
H8	0.50367	0.50580	0.23587	0.0725*	
H26B	0.38560	0.47443	0.14153	0.0855*	0.355 (5)
H27B	0.29747	0.51533	0.02799	0.1120*	0.355 (5)
H28B	0.31484	0.66227	−0.02430	0.1356*	0.355 (5)
H30B	0.50850	0.72741	0.15048	0.1040*	0.355 (5)
H33	1.24910	0.29524	0.59002	0.0740*	
H42A	0.60619	−0.09474	0.22400	0.1041*	0.626 (15)
H43A	0.65156	−0.08833	0.10594	0.1151*	0.626 (15)
H45A	0.82182	0.14934	0.12009	0.0948*	0.626 (15)
H34	1.25590	0.14401	0.63954	0.0870*	
H35	1.14792	0.03636	0.58319	0.0843*	
H36	1.03606	0.07680	0.47270	0.0701*	
H41A	0.66864	0.02090	0.29011	0.0610*	0.626 (15)
H9	1.15574	0.41003	0.47285	0.0628*	
H11C	0.90053	0.33935	0.29157	0.0602*	
H12C	0.85904	0.12812	0.29047	0.0662*	
H41B	0.77744	−0.00707	0.28610	0.0610*	0.374 (15)
H42B	0.66883	−0.11383	0.22689	0.1041*	0.374 (15)
H43B	0.59776	−0.08034	0.11108	0.1151*	0.374 (15)
H45B	0.74390	0.16667	0.11369	0.0948*	0.374 (15)

### Atomic displacement parameters (Å^2^)

**Table:**

	*U*^11^	*U*^22^	*U*^33^	*U*^12^	*U*^13^	*U*^23^
S1	0.1630 (13)	0.0644 (7)	0.0472 (7)	−0.0563 (8)	−0.0142 (7)	0.0052 (5)
F1A	0.171 (3)	0.073 (6)	0.098 (18)	−0.060 (3)	−0.026 (4)	0.009 (3)
O1	0.095 (2)	0.0627 (17)	0.0505 (18)	−0.0313 (15)	−0.0056 (16)	0.0048 (14)
N1	0.079 (2)	0.0498 (18)	0.056 (2)	−0.0262 (16)	−0.0075 (18)	0.0103 (15)
N2	0.063 (2)	0.0453 (18)	0.052 (2)	−0.0125 (15)	−0.0083 (16)	0.0004 (15)
N3	0.087 (2)	0.0456 (17)	0.043 (2)	−0.0233 (16)	−0.0071 (17)	0.0037 (14)
N4	0.081 (2)	0.0515 (18)	0.049 (2)	−0.0227 (16)	−0.0092 (17)	0.0012 (15)
C1	0.065 (3)	0.048 (2)	0.050 (3)	−0.0154 (19)	−0.001 (2)	−0.0001 (19)
C2	0.058 (3)	0.051 (2)	0.065 (3)	−0.0217 (18)	−0.004 (2)	−0.0005 (19)
C3	0.085 (3)	0.061 (3)	0.081 (4)	−0.033 (2)	−0.013 (3)	0.003 (2)
C4	0.101 (4)	0.078 (3)	0.092 (4)	−0.044 (3)	−0.028 (3)	−0.008 (3)
C5	0.106 (4)	0.084 (3)	0.073 (3)	−0.034 (3)	−0.036 (3)	0.004 (3)
C6	0.083 (3)	0.057 (2)	0.072 (3)	−0.021 (2)	−0.020 (3)	0.003 (2)
C7	0.053 (2)	0.051 (2)	0.052 (3)	−0.0123 (18)	−0.0080 (19)	0.0025 (18)
C8	0.058 (2)	0.040 (2)	0.052 (2)	−0.0130 (17)	−0.0042 (19)	0.0025 (17)
C9	0.072 (3)	0.039 (2)	0.048 (3)	−0.0150 (18)	−0.001 (2)	0.0048 (17)
C10A	0.060 (4)	0.057 (2)	0.043 (3)	−0.016 (2)	0.001 (3)	0.003 (2)
C11A	0.064 (11)	0.070 (4)	0.054 (4)	−0.026 (8)	0.007 (8)	0.002 (3)
C12A	0.166 (11)	0.101 (4)	0.057 (3)	−0.070 (7)	−0.007 (5)	0.021 (3)
C13A	0.143 (5)	0.080 (4)	0.069 (5)	−0.049 (4)	−0.011 (4)	0.025 (4)
C14A	0.017 (8)	0.042 (5)	0.073 (5)	0.007 (6)	0.001 (7)	0.000 (4)
C15A	0.036 (7)	0.051 (4)	0.050 (3)	−0.005 (5)	−0.004 (4)	0.004 (2)
C13B	0.143 (5)	0.080 (4)	0.069 (5)	−0.049 (4)	−0.011 (4)	0.025 (4)
C14B	0.017 (8)	0.042 (5)	0.073 (5)	0.007 (6)	0.001 (7)	0.000 (4)
C15B	0.036 (7)	0.051 (4)	0.050 (3)	−0.005 (5)	−0.004 (4)	0.004 (2)
C11B	0.064 (11)	0.070 (4)	0.054 (4)	−0.026 (8)	0.007 (8)	0.002 (3)
F1B	0.171 (3)	0.073 (6)	0.098 (18)	−0.060 (3)	−0.026 (4)	0.009 (3)
C10B	0.060 (4)	0.057 (2)	0.043 (3)	−0.016 (2)	0.001 (3)	0.003 (2)
C12B	0.166 (11)	0.101 (4)	0.057 (3)	−0.070 (7)	−0.007 (5)	0.021 (3)
S2	0.1054 (9)	0.0536 (6)	0.0806 (9)	−0.0286 (6)	−0.0264 (7)	0.0083 (5)
F2A	0.187 (5)	0.156 (4)	0.081 (3)	−0.042 (4)	−0.040 (3)	−0.010 (3)
O2	0.0734 (18)	0.0495 (15)	0.0530 (17)	−0.0211 (13)	−0.0128 (13)	−0.0081 (12)
N5	0.065 (2)	0.0560 (19)	0.0413 (19)	−0.0184 (16)	−0.0111 (16)	−0.0009 (15)
N6	0.059 (2)	0.0531 (18)	0.046 (2)	−0.0181 (15)	−0.0037 (16)	−0.0072 (15)
N7	0.081 (2)	0.0503 (18)	0.047 (2)	−0.0237 (16)	−0.0203 (17)	0.0018 (15)
N8	0.083 (2)	0.0583 (19)	0.043 (2)	−0.0247 (17)	−0.0149 (17)	0.0008 (16)
C16	0.046 (2)	0.049 (2)	0.047 (2)	−0.0100 (17)	−0.0018 (18)	−0.0051 (18)
C17	0.048 (2)	0.055 (2)	0.047 (3)	−0.0150 (17)	0.0027 (19)	−0.0054 (18)
C18	0.078 (3)	0.061 (3)	0.058 (3)	−0.028 (2)	0.005 (2)	0.007 (2)
C19	0.088 (3)	0.060 (3)	0.086 (4)	−0.035 (2)	0.009 (3)	−0.001 (2)
C20	0.080 (3)	0.064 (3)	0.087 (4)	−0.036 (2)	0.014 (3)	−0.025 (3)
C21	0.063 (3)	0.063 (2)	0.058 (3)	−0.026 (2)	0.002 (2)	−0.017 (2)
C22	0.046 (2)	0.050 (2)	0.050 (2)	−0.0155 (17)	0.0051 (19)	−0.0066 (18)
C23	0.049 (2)	0.049 (2)	0.037 (2)	−0.0123 (17)	−0.0034 (18)	−0.0076 (17)
C24	0.057 (2)	0.053 (2)	0.053 (3)	−0.0131 (18)	−0.006 (2)	−0.001 (2)
C25A	0.066 (3)	0.070 (3)	0.042 (3)	−0.013 (2)	−0.002 (2)	0.005 (2)
C26A	0.074 (3)	0.082 (3)	0.049 (3)	−0.001 (2)	−0.014 (2)	−0.009 (2)
C27A	0.096 (4)	0.112 (4)	0.062 (4)	−0.004 (3)	−0.019 (3)	−0.024 (3)
C28A	0.126 (5)	0.141 (6)	0.052 (3)	−0.001 (4)	−0.001 (3)	0.014 (3)
C29A	0.136 (5)	0.100 (4)	0.091 (5)	−0.023 (4)	0.001 (4)	0.030 (3)
C30A	0.106 (4)	0.100 (4)	0.055 (3)	−0.034 (3)	−0.007 (3)	0.021 (3)
C28B	0.126 (5)	0.141 (6)	0.052 (3)	−0.001 (4)	−0.001 (3)	0.014 (3)
C29B	0.136 (5)	0.100 (4)	0.091 (5)	−0.023 (4)	0.001 (4)	0.030 (3)
C30B	0.106 (4)	0.100 (4)	0.055 (3)	−0.034 (3)	−0.007 (3)	0.021 (3)
C26B	0.074 (3)	0.082 (3)	0.049 (3)	−0.001 (2)	−0.014 (2)	−0.009 (2)
F2B	0.187 (5)	0.156 (4)	0.081 (3)	−0.042 (4)	−0.040 (3)	−0.010 (3)
C25B	0.066 (3)	0.070 (3)	0.042 (3)	−0.013 (2)	−0.002 (2)	0.005 (2)
C27B	0.096 (4)	0.112 (4)	0.062 (4)	−0.004 (3)	−0.019 (3)	−0.024 (3)
S3	0.1131 (10)	0.0550 (6)	0.0586 (8)	−0.0233 (6)	−0.0331 (7)	0.0093 (5)
F3A	0.292 (18)	0.175 (4)	0.063 (3)	−0.122 (11)	−0.021 (8)	−0.038 (3)
O3	0.088 (2)	0.0521 (15)	0.0539 (18)	−0.0302 (14)	−0.0128 (14)	0.0043 (13)
N9	0.063 (2)	0.0483 (18)	0.051 (2)	−0.0230 (15)	−0.0094 (16)	−0.0048 (15)
N10	0.0542 (19)	0.0480 (17)	0.0428 (19)	−0.0165 (14)	−0.0097 (15)	0.0037 (14)
N11	0.067 (2)	0.0447 (16)	0.0403 (19)	−0.0158 (14)	−0.0100 (16)	−0.0016 (14)
N12	0.077 (2)	0.0442 (17)	0.046 (2)	−0.0190 (15)	−0.0147 (16)	0.0039 (14)
C31	0.053 (2)	0.049 (2)	0.047 (2)	−0.0159 (17)	0.0005 (19)	−0.0039 (18)
C32	0.045 (2)	0.053 (2)	0.046 (2)	−0.0153 (17)	−0.0051 (18)	−0.0034 (18)
C33	0.072 (3)	0.065 (3)	0.053 (3)	−0.025 (2)	−0.014 (2)	−0.006 (2)
C34	0.095 (3)	0.076 (3)	0.048 (3)	−0.025 (2)	−0.024 (2)	0.009 (2)
C35	0.098 (3)	0.057 (2)	0.057 (3)	−0.025 (2)	−0.018 (2)	0.010 (2)
C36	0.074 (3)	0.053 (2)	0.053 (3)	−0.0247 (19)	−0.010 (2)	−0.0001 (19)
C37	0.049 (2)	0.044 (2)	0.044 (2)	−0.0145 (16)	−0.0085 (18)	−0.0027 (17)
C38	0.046 (2)	0.046 (2)	0.042 (2)	−0.0139 (16)	−0.0029 (18)	−0.0028 (17)
C39	0.053 (2)	0.054 (2)	0.042 (2)	−0.0142 (18)	−0.0074 (18)	−0.0043 (17)
C40A	0.057 (4)	0.047 (3)	0.047 (3)	−0.008 (2)	−0.010 (3)	−0.008 (2)
C41A	0.025 (7)	0.045 (5)	0.076 (3)	0.002 (5)	−0.006 (5)	−0.001 (3)
C42A	0.087 (6)	0.056 (4)	0.122 (5)	−0.026 (4)	−0.022 (5)	−0.006 (3)
C43A	0.099 (7)	0.071 (4)	0.118 (5)	−0.013 (4)	−0.040 (5)	−0.034 (3)
C44A	0.090 (10)	0.082 (6)	0.060 (4)	−0.004 (8)	−0.035 (6)	−0.017 (4)
C45A	0.115 (9)	0.065 (4)	0.058 (4)	−0.025 (5)	−0.025 (5)	−0.006 (3)
C43B	0.099 (7)	0.071 (4)	0.118 (5)	−0.013 (4)	−0.040 (5)	−0.034 (3)
C44B	0.090 (10)	0.082 (6)	0.060 (4)	−0.004 (8)	−0.035 (6)	−0.017 (4)
C45B	0.115 (9)	0.065 (4)	0.058 (4)	−0.025 (5)	−0.025 (5)	−0.006 (3)
C41B	0.025 (7)	0.045 (5)	0.076 (3)	0.002 (5)	−0.006 (5)	−0.001 (3)
F3B	0.292 (18)	0.175 (4)	0.063 (3)	−0.122 (11)	−0.021 (8)	−0.038 (3)
C40B	0.057 (4)	0.047 (3)	0.047 (3)	−0.008 (2)	−0.010 (3)	−0.008 (2)
C42B	0.087 (6)	0.056 (4)	0.122 (5)	−0.026 (4)	−0.022 (5)	−0.006 (3)

### Geometric parameters (Å, °)

**Table:**

S1—C9	1.641 (4)	C12A—H12A	0.9300
S2—C24	1.650 (4)	C12B—H12B	0.9300
S3—C39	1.651 (4)	C13A—H13A	0.9300
F1A—C14A	1.35 (12)	C13B—H13B	0.9300
F1B—C14B	1.36 (16)	C15A—H15A	0.9300
F2A—C27A	1.356 (6)	C15B—H15B	0.9300
F2B—C29B	1.331 (5)	C16—C23	1.495 (5)
F3A—C44A	1.344 (18)	C17—C18	1.376 (6)
F3B—C44B	1.66 (4)	C17—C22	1.394 (5)
O1—C1	1.217 (5)	C18—C19	1.376 (6)
O2—C16	1.238 (5)	C19—C20	1.377 (8)
O3—C31	1.241 (4)	C20—C21	1.382 (6)
N1—C1	1.343 (5)	C21—C22	1.374 (6)
N1—C2	1.398 (5)	C22—C23	1.449 (5)
N2—C8	1.288 (4)	C25A—C30A	1.389 (6)
N2—N3	1.353 (4)	C25A—C26A	1.390 (6)
N3—C9	1.366 (4)	C25B—C26B	1.390 (4)
N4—C9	1.341 (5)	C25B—C30B	1.390 (4)
N4—C10B	1.38 (2)	C26A—C27A	1.390 (5)
N4—C10A	1.432 (16)	C26B—C27B	1.390 (5)
N1—H1	0.8600	C27A—C28A	1.390 (6)
N3—H3A	0.8600	C27B—C28B	1.390 (4)
N4—H4A	0.8600	C28A—C29A	1.390 (7)
N5—C16	1.338 (5)	C28B—C29B	1.390 (6)
N5—C17	1.410 (5)	C29A—C30A	1.390 (8)
N6—C23	1.295 (5)	C29B—C30B	1.390 (6)
N6—N7	1.338 (4)	C18—H18	0.9300
N7—C24	1.390 (5)	C19—H19	0.9300
N8—C25B	1.396 (5)	C20—H20	0.9300
N8—C24	1.328 (5)	C21—H21	0.9300
N8—C25A	1.391 (5)	C26A—H26A	0.9300
N5—H5A	0.8600	C26B—H26B	0.9300
N7—H7	0.8600	C27B—H27B	0.9300
N8—H8	0.8600	C28A—H28A	0.9300
N9—C31	1.340 (4)	C28B—H28B	0.9300
N9—C32	1.413 (4)	C29A—H29A	0.9300
N10—C38	1.287 (4)	C30A—H30A	0.9300
N10—N11	1.351 (4)	C30B—H30B	0.9300
N11—C39	1.370 (5)	C31—C38	1.496 (5)
N12—C40B	1.45 (2)	C32—C37	1.397 (5)
N12—C39	1.331 (5)	C32—C33	1.362 (5)
N12—C40A	1.379 (13)	C33—C34	1.372 (6)
N9—H9	0.8600	C34—C35	1.384 (6)
N11—H11C	0.8600	C35—C36	1.382 (6)
N12—H12C	0.8600	C36—C37	1.385 (5)
C1—C8	1.505 (5)	C37—C38	1.442 (5)
C2—C3	1.368 (6)	C40A—C41A	1.390 (17)
C2—C7	1.392 (6)	C40A—C45A	1.391 (19)
C3—C4	1.369 (8)	C40B—C41B	1.39 (3)
C4—C5	1.375 (7)	C40B—C45B	1.39 (4)
C5—C6	1.391 (6)	C41A—C42A	1.390 (18)
C6—C7	1.374 (6)	C41B—C42B	1.39 (3)
C7—C8	1.443 (5)	C42A—C43A	1.39 (2)
C10A—C15A	1.39 (2)	C42B—C43B	1.39 (4)
C10A—C11A	1.39 (3)	C43A—C44A	1.39 (2)
C10B—C15B	1.39 (3)	C43B—C44B	1.40 (4)
C10B—C11B	1.39 (4)	C44A—C45A	1.39 (2)
C11A—C12A	1.39 (3)	C44B—C45B	1.39 (3)
C11B—C12B	1.39 (3)	C33—H33	0.9300
C12A—C13A	1.39 (3)	C34—H34	0.9300
C12B—C13B	1.39 (3)	C35—H35	0.9300
C13A—C14A	1.39 (3)	C36—H36	0.9300
C13B—C14B	1.39 (4)	C41A—H41A	0.9300
C14A—C15A	1.39 (2)	C41B—H41B	0.9300
C14B—C15B	1.39 (3)	C42A—H42A	0.9300
C3—H3	0.9300	C42B—H42B	0.9300
C4—H4	0.9300	C43A—H43A	0.9300
C5—H5	0.9300	C43B—H43B	0.9300
C6—H6	0.9300	C45A—H45A	0.9300
C11A—H11A	0.9300	C45B—H45B	0.9300
C11B—H11B	0.9300		
			
S1···C15A	3.223 (18)	C23···C38	3.565 (4)
S1···C15B	3.20 (2)	C23···N11	3.406 (4)
S2···C30A	3.208 (6)	C23···C31	3.568 (5)
S2···N3	3.454 (3)	C24···O1	3.401 (5)
S2···C30B	3.195 (4)	C24···C1	3.471 (6)
S2···C33^i^	3.618 (4)	C25A···C7	3.473 (6)
S3···C26A	3.646 (4)	C25A···C8	3.559 (6)
S3···C26B	3.653 (3)	C25B···C8	3.577 (5)
S3···C45B	3.23 (2)	C25B···C7	3.498 (6)
S3···C45A	3.260 (13)	C26A···C2	3.501 (6)
S1···H20^ii^	2.9500	C26A···C7	3.524 (6)
S1···H18^iii^	2.9900	C26A···S3	3.646 (4)
S1···H15B	2.5400	C26B···C7	3.532 (5)
S1···H15A	2.5700	C26B···C2	3.521 (5)
S2···H42A^ii^	2.8700	C26B···S3	3.653 (3)
S2···H33^i^	2.9200	C27A···C6	3.511 (6)
S2···H42B^ii^	2.8000	C27A···C27A^v^	3.424 (5)
S2···H30B	2.5300	C27A···C5	3.406 (6)
S2···H34^i^	3.1800	C27B···C5	3.415 (6)
S2···H30A	2.5400	C27B···C6	3.507 (5)
S3···H26B	3.1500	C27B···C27B^v^	3.421 (5)
S3···H45A	2.7200	C28A···C6	3.542 (7)
S3···H3^iv^	3.1400	C28A···F2A^v^	3.314 (7)
S3···H45B	2.6200	C28B···C6	3.551 (7)
S3···H28A^v^	2.9600	C30A···S2	3.208 (6)
S3···H8	3.1000	C30A···C5^iv^	3.597 (8)
S3···H26A	3.1500	C30B···C4^iv^	3.588 (7)
S3···H28B^v^	2.9700	C30B···S2	3.195 (4)
F2A···C4^vi^	3.215 (7)	C30B···C5^iv^	3.567 (8)
F2A···C28A^v^	3.314 (7)	C31···C23	3.568 (5)
F2B···F3A^v^	2.534 (17)	C31···C16	3.247 (5)
F3A···F2B^v^	2.534 (17)	C32···C20^iv^	3.507 (6)
F3B···C11A^v^	3.30 (3)	C32···C19^iv^	3.531 (6)
F1A···H21^ii^	2.6200	C33···C19^iv^	3.484 (7)
F1B···H21^ii^	2.7100	C33···O1^iii^	3.305 (5)
F2A···H4^vi^	2.4800	C33···C18^iv^	3.472 (6)
F2B···H12A^vii^	2.8000	C33···S2^i^	3.618 (4)
F3B···H11A^v^	2.4600	C34···O1^iii^	3.405 (5)
O1···C24	3.401 (5)	C36···C18	3.510 (6)
O1···N3	2.778 (4)	C37···N5	3.428 (4)
O1···C34^iii^	3.405 (5)	C37···C20^iv^	3.539 (5)
O1···N5^iii^	2.881 (4)	C37···C17	3.500 (5)
O1···N2	3.028 (4)	C38···C16	3.552 (5)
O1···C33^iii^	3.305 (5)	C38···C22	3.510 (5)
O2···N6	3.032 (4)	C38···N5	3.431 (4)
O2···N7	2.785 (4)	C38···C23	3.565 (4)
O2···C16^iii^	3.220 (4)	C38···C17	3.420 (5)
O2···N9^i^	2.871 (3)	C39···C21	3.587 (5)
O3···N11	2.767 (3)	C39···C15A^x^	3.280 (16)
O3···N1^iv^	2.837 (3)	C39···C14A^x^	3.565 (16)
O3···N10	3.020 (3)	C40B···N4^x^	3.31 (2)
O1···H5A^iii^	2.0500	C41B···N2^x^	3.37 (2)
O1···H3A	2.1100	C41B···C9^x^	3.594 (18)
O2···H7	2.1100	C42B···N2^x^	3.39 (2)
O2···H9^i^	2.0600	C43B···C10A^xi^	3.41 (3)
O3···H11C	2.0900	C43B···C11A^xi^	3.23 (3)
O3···H1^iv^	2.0300	C44B···C11A^xi^	3.28 (3)
O3···H3^iv^	2.9200	C44B···C12A^xi^	3.27 (3)
N1···O3^viii^	2.837 (3)	C45A···S3	3.260 (13)
N2···O1	3.028 (4)	C45B···S3	3.23 (2)
N2···N4	2.596 (4)	C1···H3A	2.4800
N2···C41B^ix^	3.37 (2)	C9···H15A	2.8400
N2···C42B^ix^	3.39 (2)	C9···H15B	2.9000
N3···O1	2.778 (4)	C16···H7	2.4900
N3···S2	3.454 (3)	C24···H30A	2.8500
N4···C40B^ix^	3.31 (2)	C24···H30B	2.8600
N4···N2	2.596 (4)	C31···H11C	2.4900
N5···C38	3.431 (4)	C34···H41B^xii^	3.0800
N5···O1^iii^	2.881 (4)	C39···H45B	2.9400
N5···C37	3.428 (4)	C39···H45A	2.8300
N6···O2	3.032 (4)	C41B···H34^xii^	3.0900
N6···N8	2.599 (4)	H1···O3^viii^	2.0300
N7···O2	2.785 (4)	H3···O3^viii^	2.9200
N8···N6	2.599 (4)	H3···S3^viii^	3.1400
N9···O2^i^	2.871 (3)	H3···H11C^viii^	2.5700
N10···N12	2.590 (4)	H3A···C1	2.4800
N10···C22	3.248 (5)	H3A···O1	2.1100
N10···O3	3.020 (3)	H4···F2A^vi^	2.4800
N11···C22	3.426 (5)	H4A···H11A	2.3800
N11···O3	2.767 (3)	H4A···H11B	2.1100
N11···C15A^x^	3.307 (17)	H4A···N2	2.1300
N11···C23	3.406 (4)	H5A···O1^iii^	2.0500
N12···N10	2.590 (4)	H7···H33^i^	2.5100
N12···C15A^x^	3.348 (16)	H7···O2	2.1100
N12···C10B^x^	3.399 (19)	H7···C16	2.4900
N2···H4A	2.1300	H8···S3	3.1000
N6···H8	2.1400	H8···N6	2.1400
N10···H15A^x^	2.8000	H8···H26B	2.2000
N10···H12C	2.1300	H8···H26A	2.2000
N11···H15A^x^	2.9100	H9···O2^i^	2.0600
C1···C24	3.471 (6)	H11A···H4A	2.3800
C2···C26A	3.501 (6)	H11A···F3B^v^	2.4600
C2···C26B	3.521 (5)	H11B···H4A	2.1100
C4···F2A^vi^	3.215 (7)	H11C···C31	2.4900
C4···C30B^viii^	3.588 (7)	H11C···H3^iv^	2.5700
C5···C27A	3.406 (6)	H11C···O3	2.0900
C5···C30B^viii^	3.567 (8)	H12A···F2B^vii^	2.8000
C5···C30A^viii^	3.597 (8)	H12C···H41A	2.4400
C5···C27B	3.415 (6)	H12C···H41B	2.2100
C6···C27A	3.511 (6)	H12C···N10	2.1300
C6···C27B	3.507 (5)	H15A···N11^ix^	2.9100
C6···C28B	3.551 (7)	H15A···S1	2.5700
C6···C28A	3.542 (7)	H15A···C9	2.8400
C7···C26B	3.532 (5)	H15A···N10^ix^	2.8000
C7···C26A	3.524 (6)	H15B···C9	2.9000
C7···C25A	3.473 (6)	H15B···S1	2.5400
C7···C25B	3.498 (6)	H18···S1^iii^	2.9900
C8···C25A	3.559 (6)	H20···S1^xi^	2.9500
C8···C25B	3.577 (5)	H21···F1B^xi^	2.7100
C9···C41B^ix^	3.594 (18)	H21···F1A^xi^	2.6200
C10A···C43B^ii^	3.41 (3)	H26A···H8	2.2000
C10B···N12^ix^	3.399 (19)	H26A···S3	3.1500
C11A···C43B^ii^	3.23 (3)	H26B···S3	3.1500
C11A···F3B^v^	3.30 (3)	H26B···H8	2.2000
C11A···C44B^ii^	3.28 (3)	H28A···S3^v^	2.9600
C12A···C44B^ii^	3.27 (3)	H28B···S3^v^	2.9700
C14A···C39^ix^	3.565 (16)	H30A···C24	2.8500
C15A···C39^ix^	3.280 (16)	H30A···S2	2.5400
C15A···N12^ix^	3.348 (16)	H30B···C24	2.8600
C15A···S1	3.223 (18)	H30B···S2	2.5300
C15A···N11^ix^	3.307 (17)	H33···H7^i^	2.5100
C15B···S1	3.20 (2)	H33···S2^i^	2.9200
C16···O2^iii^	3.220 (4)	H34···S2^i^	3.1800
C16···C38	3.552 (5)	H34···H41A^xii^	2.5900
C16···C31	3.247 (5)	H34···C41B^xii^	3.0900
C16···C16^iii^	3.217 (5)	H34···H41B^xii^	2.4400
C17···C38	3.420 (5)	H36···H36^xii^	2.5800
C17···C37	3.500 (5)	H41A···H34^xii^	2.5900
C18···C33^viii^	3.472 (6)	H41A···H12C	2.4400
C18···C36	3.510 (6)	H41B···C34^xii^	3.0800
C19···C32^viii^	3.531 (6)	H41B···H34^xii^	2.4400
C19···C33^viii^	3.484 (7)	H41B···H12C	2.2100
C20···C32^viii^	3.507 (6)	H42A···S2^xi^	2.8700
C20···C37^viii^	3.539 (5)	H42B···S2^xi^	2.8000
C21···C39	3.587 (5)	H45A···S3	2.7200
C22···C38	3.510 (5)	H45A···C39	2.8300
C22···N10	3.248 (5)	H45B···S3	2.6200
C22···N11	3.426 (5)	H45B···C39	2.9400
			
C1—N1—C2	111.9 (3)	C16—C23—C22	107.0 (3)
N3—N2—C8	117.6 (3)	N6—C23—C22	124.8 (3)
N2—N3—C9	121.8 (3)	S2—C24—N7	116.8 (3)
C9—N4—C10A	131.9 (8)	S2—C24—N8	130.1 (3)
C9—N4—C10B	130.5 (10)	N7—C24—N8	113.1 (3)
C1—N1—H1	124.00	N8—C25A—C30A	124.4 (4)
C2—N1—H1	124.00	N8—C25A—C26A	115.6 (3)
N2—N3—H3A	119.00	C26A—C25A—C30A	120.0 (4)
C9—N3—H3A	119.00	C26B—C25B—C30B	120.0 (3)
C10B—N4—H4A	115.00	N8—C25B—C26B	114.9 (2)
C9—N4—H4A	114.00	N8—C25B—C30B	125.2 (3)
C10A—N4—H4A	114.00	C25A—C26A—C27A	120.0 (4)
C16—N5—C17	111.7 (3)	C25B—C26B—C27B	120.0 (3)
N7—N6—C23	117.9 (3)	F2A—C27A—C26A	117.6 (4)
N6—N7—C24	120.8 (3)	C26A—C27A—C28A	120.0 (4)
C24—N8—C25A	132.0 (3)	F2A—C27A—C28A	122.3 (3)
C24—N8—C25B	131.1 (3)	C26B—C27B—C28B	120.0 (3)
C17—N5—H5A	124.00	C27A—C28A—C29A	120.0 (4)
C16—N5—H5A	124.00	C27B—C28B—C29B	120.0 (3)
C24—N7—H7	120.00	C28A—C29A—C30A	120.0 (4)
N6—N7—H7	120.00	F2B—C29B—C30B	112.7 (4)
C24—N8—H8	114.00	F2B—C29B—C28B	127.3 (4)
C25A—N8—H8	114.00	C28B—C29B—C30B	120.0 (3)
C25B—N8—H8	115.00	C25A—C30A—C29A	120.0 (5)
C31—N9—C32	111.3 (3)	C25B—C30B—C29B	120.0 (4)
N11—N10—C38	117.8 (3)	C19—C18—H18	122.00
N10—N11—C39	120.8 (3)	C17—C18—H18	122.00
C39—N12—C40B	130.4 (10)	C18—C19—H19	119.00
C39—N12—C40A	130.8 (6)	C20—C19—H19	119.00
C31—N9—H9	124.00	C21—C20—H20	120.00
C32—N9—H9	124.00	C19—C20—H20	120.00
C39—N11—H11C	120.00	C20—C21—H21	121.00
N10—N11—H11C	120.00	C22—C21—H21	121.00
C40B—N12—H12C	114.00	C27A—C26A—H26A	120.00
C39—N12—H12C	115.00	C25A—C26A—H26A	120.00
C40A—N12—H12C	115.00	C27B—C26B—H26B	120.00
O1—C1—N1	127.4 (4)	C25B—C26B—H26B	120.00
O1—C1—C8	126.9 (4)	C28B—C27B—H27B	120.00
N1—C1—C8	105.6 (3)	C26B—C27B—H27B	120.00
C3—C2—C7	121.7 (4)	C29A—C28A—H28A	120.00
N1—C2—C3	128.9 (4)	C27A—C28A—H28A	120.00
N1—C2—C7	109.3 (3)	C27B—C28B—H28B	120.00
C2—C3—C4	117.9 (4)	C29B—C28B—H28B	120.00
C3—C4—C5	121.4 (5)	C28A—C29A—H29A	120.00
C4—C5—C6	120.9 (5)	C30A—C29A—H29A	120.00
C5—C6—C7	118.0 (4)	C29A—C30A—H30A	120.00
C2—C7—C8	106.8 (3)	C25A—C30A—H30A	120.00
C2—C7—C6	120.2 (4)	C25B—C30B—H30B	120.00
C6—C7—C8	133.1 (4)	C29B—C30B—H30B	120.00
N2—C8—C7	125.7 (3)	O3—C31—N9	127.5 (3)
N2—C8—C1	127.9 (3)	O3—C31—C38	126.0 (3)
C1—C8—C7	106.4 (3)	N9—C31—C38	106.6 (3)
S1—C9—N4	129.3 (3)	N9—C32—C33	128.5 (3)
N3—C9—N4	112.6 (3)	N9—C32—C37	108.9 (3)
S1—C9—N3	118.1 (3)	C33—C32—C37	122.6 (4)
C11A—C10A—C15A	120.0 (15)	C32—C33—C34	117.2 (4)
N4—C10A—C11A	118.8 (14)	C33—C34—C35	122.2 (4)
N4—C10A—C15A	120.7 (15)	C34—C35—C36	120.0 (4)
N4—C10B—C11B	112.3 (17)	C35—C36—C37	118.8 (4)
C11B—C10B—C15B	120.1 (19)	C32—C37—C36	119.2 (3)
N4—C10B—C15B	127 (2)	C36—C37—C38	133.8 (3)
C10A—C11A—C12A	120.0 (16)	C32—C37—C38	107.0 (3)
C10B—C11B—C12B	120 (2)	N10—C38—C37	125.5 (3)
C11A—C12A—C13A	120.0 (17)	C31—C38—C37	106.4 (3)
C11B—C12B—C13B	120 (2)	N10—C38—C31	128.2 (3)
C12A—C13A—C14A	120.0 (15)	S3—C39—N11	117.9 (3)
C12B—C13B—C14B	120.1 (19)	S3—C39—N12	128.8 (3)
F1A—C14A—C15A	121 (6)	N11—C39—N12	113.3 (3)
F1A—C14A—C13A	116 (6)	N12—C40A—C41A	119.5 (11)
C13A—C14A—C15A	120.0 (15)	N12—C40A—C45A	119.7 (10)
F1B—C14B—C13B	119 (7)	C41A—C40A—C45A	120.0 (11)
C13B—C14B—C15B	120.0 (18)	N12—C40B—C45B	126.4 (18)
F1B—C14B—C15B	115 (7)	N12—C40B—C41B	112.7 (19)
C10A—C15A—C14A	120.1 (16)	C41B—C40B—C45B	120 (2)
C10B—C15B—C14B	120 (2)	C40A—C41A—C42A	120.0 (12)
C4—C3—H3	121.00	C40B—C41B—C42B	120 (2)
C2—C3—H3	121.00	C41A—C42A—C43A	120.0 (12)
C3—C4—H4	119.00	C41B—C42B—C43B	120 (2)
C5—C4—H4	119.00	C42A—C43A—C44A	120.0 (12)
C4—C5—H5	120.00	C42B—C43B—C44B	120 (2)
C6—C5—H5	120.00	C43A—C44A—C45A	120.0 (12)
C7—C6—H6	121.00	F3A—C44A—C43A	107.4 (14)
C5—C6—H6	121.00	F3A—C44A—C45A	125.4 (15)
C10A—C11A—H11A	120.00	F3B—C44B—C45B	98 (2)
C12A—C11A—H11A	120.00	F3B—C44B—C43B	122 (2)
C10B—C11B—H11B	120.00	C43B—C44B—C45B	120 (2)
C12B—C11B—H11B	120.00	C40A—C45A—C44A	120.0 (12)
C11A—C12A—H12A	120.00	C40B—C45B—C44B	120 (2)
C13A—C12A—H12A	120.00	C32—C33—H33	121.00
C13B—C12B—H12B	120.00	C34—C33—H33	121.00
C11B—C12B—H12B	120.00	C33—C34—H34	119.00
C14A—C13A—H13A	120.00	C35—C34—H34	119.00
C12A—C13A—H13A	120.00	C34—C35—H35	120.00
C14B—C13B—H13B	120.00	C36—C35—H35	120.00
C12B—C13B—H13B	120.00	C35—C36—H36	121.00
C10A—C15A—H15A	120.00	C37—C36—H36	121.00
C14A—C15A—H15A	120.00	C40A—C41A—H41A	120.00
C14B—C15B—H15B	120.00	C42A—C41A—H41A	120.00
C10B—C15B—H15B	120.00	C42B—C41B—H41B	120.00
N5—C16—C23	105.9 (3)	C40B—C41B—H41B	120.00
O2—C16—N5	127.6 (3)	C43A—C42A—H42A	120.00
O2—C16—C23	126.5 (3)	C41A—C42A—H42A	120.00
N5—C17—C22	109.3 (3)	C43B—C42B—H42B	120.00
N5—C17—C18	128.6 (3)	C41B—C42B—H42B	120.00
C18—C17—C22	122.1 (4)	C44A—C43A—H43A	120.00
C17—C18—C19	116.5 (4)	C42A—C43A—H43A	120.00
C18—C19—C20	122.3 (4)	C44B—C43B—H43B	120.00
C19—C20—C21	120.8 (4)	C42B—C43B—H43B	120.00
C20—C21—C22	118.0 (4)	C44A—C45A—H45A	120.00
C21—C22—C23	133.5 (4)	C40A—C45A—H45A	120.00
C17—C22—C23	106.1 (3)	C40B—C45B—H45B	120.00
C17—C22—C21	120.4 (4)	C44B—C45B—H45B	120.00
N6—C23—C16	128.2 (3)		
			
C2—N1—C1—O1	−178.5 (4)	C11A—C12A—C13A—C14A	0(2)
C2—N1—C1—C8	−0.4 (4)	C12A—C13A—C14A—F1A	161 (4)
C1—N1—C2—C3	−179.8 (4)	C12A—C13A—C14A—C15A	0(2)
C1—N1—C2—C7	0.0 (5)	C13A—C14A—C15A—C10A	0(2)
C8—N2—N3—C9	173.8 (3)	F1A—C14A—C15A—C10A	−160 (4)
N3—N2—C8—C1	−2.7 (5)	O2—C16—C23—N6	2.3 (6)
N3—N2—C8—C7	178.0 (3)	N5—C16—C23—N6	−176.6 (3)
N2—N3—C9—S1	178.8 (3)	N5—C16—C23—C22	1.7 (4)
N2—N3—C9—N4	−1.0 (5)	O2—C16—C23—C22	−179.5 (3)
C10A—N4—C9—S1	2.8 (10)	N5—C17—C18—C19	−178.1 (4)
C10A—N4—C9—N3	−177.4 (9)	C18—C17—C22—C23	−178.0 (3)
C9—N4—C10A—C11A	161.3 (10)	N5—C17—C22—C21	178.2 (3)
C9—N4—C10A—C15A	−26.6 (19)	N5—C17—C22—C23	−0.1 (4)
C16—N5—C17—C18	179.0 (4)	C22—C17—C18—C19	−0.6 (6)
C16—N5—C17—C22	1.2 (4)	C18—C17—C22—C21	0.3 (6)
C17—N5—C16—C23	−1.8 (4)	C17—C18—C19—C20	0.9 (7)
C17—N5—C16—O2	179.4 (4)	C18—C19—C20—C21	−0.9 (7)
N7—N6—C23—C16	−1.7 (5)	C19—C20—C21—C22	0.5 (6)
C23—N6—N7—C24	179.6 (3)	C20—C21—C22—C17	−0.2 (6)
N7—N6—C23—C22	−179.7 (3)	C20—C21—C22—C23	177.6 (4)
N6—N7—C24—N8	−6.0 (5)	C17—C22—C23—N6	177.4 (3)
N6—N7—C24—S2	173.4 (3)	C17—C22—C23—C16	−0.9 (4)
C24—N8—C25A—C26A	−172.7 (4)	C21—C22—C23—C16	−178.9 (4)
C24—N8—C25A—C30A	8.5 (8)	C21—C22—C23—N6	−0.6 (7)
C25A—N8—C24—S2	−1.6 (7)	C30A—C25A—C26A—C27A	0.0 (7)
C25A—N8—C24—N7	177.7 (4)	N8—C25A—C26A—C27A	−178.9 (4)
C31—N9—C32—C37	0.3 (4)	C26A—C25A—C30A—C29A	0.0 (8)
C31—N9—C32—C33	−179.2 (4)	N8—C25A—C30A—C29A	178.9 (5)
C32—N9—C31—O3	−179.6 (3)	C25A—C26A—C27A—F2A	−176.5 (4)
C32—N9—C31—C38	−0.3 (4)	C25A—C26A—C27A—C28A	0.0 (6)
N11—N10—C38—C31	−1.1 (5)	C26A—C27A—C28A—C29A	0.0 (7)
C38—N10—N11—C39	178.7 (3)	F2A—C27A—C28A—C29A	176.3 (5)
N11—N10—C38—C37	177.1 (3)	C27A—C28A—C29A—C30A	0.0 (9)
N10—N11—C39—S3	176.3 (3)	C28A—C29A—C30A—C25A	0.0 (9)
N10—N11—C39—N12	−3.4 (5)	O3—C31—C38—N10	−1.9 (6)
C40A—N12—C39—S3	0.2 (9)	O3—C31—C38—C37	179.6 (3)
C39—N12—C40A—C45A	41.9 (14)	N9—C31—C38—N10	178.8 (3)
C40A—N12—C39—N11	180.0 (8)	N9—C31—C38—C37	0.3 (4)
C39—N12—C40A—C41A	−148.3 (8)	N9—C32—C33—C34	180.0 (4)
N1—C1—C8—C7	0.6 (4)	C37—C32—C33—C34	0.6 (6)
N1—C1—C8—N2	−178.7 (4)	N9—C32—C37—C36	−179.6 (3)
O1—C1—C8—N2	−0.6 (7)	N9—C32—C37—C38	−0.1 (4)
O1—C1—C8—C7	178.8 (4)	C33—C32—C37—C36	−0.1 (6)
C7—C2—C3—C4	−0.1 (7)	C33—C32—C37—C38	179.5 (3)
N1—C2—C3—C4	179.7 (4)	C32—C33—C34—C35	−0.9 (7)
N1—C2—C7—C6	179.8 (4)	C33—C34—C35—C36	0.8 (7)
N1—C2—C7—C8	0.4 (5)	C34—C35—C36—C37	−0.4 (6)
C3—C2—C7—C8	−179.8 (4)	C35—C36—C37—C32	0.0 (6)
C3—C2—C7—C6	−0.4 (6)	C35—C36—C37—C38	−179.4 (4)
C2—C3—C4—C5	0.5 (7)	C32—C37—C38—N10	−178.7 (3)
C3—C4—C5—C6	−0.4 (7)	C32—C37—C38—C31	−0.1 (4)
C4—C5—C6—C7	−0.1 (7)	C36—C37—C38—N10	0.8 (6)
C5—C6—C7—C8	179.6 (4)	C36—C37—C38—C31	179.3 (4)
C5—C6—C7—C2	0.5 (6)	N12—C40A—C41A—C42A	−169.7 (10)
C2—C7—C8—N2	178.8 (4)	C45A—C40A—C41A—C42A	0.0 (18)
C6—C7—C8—C1	−179.9 (5)	N12—C40A—C45A—C44A	169.7 (11)
C6—C7—C8—N2	−0.5 (7)	C41A—C40A—C45A—C44A	0.0 (19)
C2—C7—C8—C1	−0.7 (4)	C40A—C41A—C42A—C43A	0.0 (18)
C11A—C10A—C15A—C14A	0(2)	C41A—C42A—C43A—C44A	0(2)
N4—C10A—C15A—C14A	−172.1 (13)	C42A—C43A—C44A—F3A	−152.0 (14)
C15A—C10A—C11A—C12A	0(2)	C42A—C43A—C44A—C45A	0(2)
N4—C10A—C11A—C12A	172.5 (14)	F3A—C44A—C45A—C40A	146.6 (15)
C10A—C11A—C12A—C13A	−1(3)	C43A—C44A—C45A—C40A	0(2)

Symmetry codes: (i) −*x*+2, −*y*+1, −*z*+1; (ii) *x*, *y*+1, *z*; (iii) −*x*+1, −*y*+1, −*z*+1; (iv) *x*+1, *y*, *z*; (v) −*x*+1, −*y*+1, −*z*; (vi) −*x*, −*y*+1, −*z*; (vii) −*x*+1, −*y*+2, −*z*; (viii) *x*−1, *y*, *z*; (ix) *x*−1, *y*+1, *z*; (x) *x*+1, *y*−1, *z*; (xi) *x*, *y*−1, *z*; (xii) −*x*+2, −*y*, −*z*+1.

### Hydrogen-bond geometry (Å, °)

**Table:**

*D*—H···*A*	*D*—H	H···*A*	*D*···*A*	*D*—H···*A*
N1—H1···O3^viii^	0.86	2.03	2.837 (3)	155
N3—H3A···O1	0.86	2.11	2.778 (4)	134
N4—H4A···N2	0.86	2.13	2.596 (4)	114
N5—H5A···O1^iii^	0.86	2.05	2.881 (4)	164
N7—H7···O2	0.86	2.11	2.785 (4)	135
N8—H8···N6	0.86	2.14	2.599 (4)	113
N9—H9···O2^i^	0.86	2.06	2.871 (3)	157
N11—H11C···O3	0.86	2.09	2.767 (3)	135
N12—H12C···N10	0.86	2.13	2.590 (4)	113
C4—H4···F2A^vi^	0.93	2.48	3.215 (7)	136
C15A—H15A···S1	0.93	2.57	3.223 (18)	127
C30A—H30A···S2	0.93	2.54	3.208 (6)	129
C42A—H42A···S2^xi^	0.93	2.87	3.763 (12)	162
C45A—H45A···S3	0.93	2.72	3.260 (13)	118

Symmetry codes: (viii) *x*−1, *y*, *z*; (iii) −*x*+1, −*y*+1, −*z*+1; (i) −*x*+2, −*y*+1, −*z*+1; (vi) −*x*, −*y*+1, −*z*; (xi) *x*, *y*−1, *z*.
